# Next Generation dsRNA-Based Insect Control: Success So Far and Challenges

**DOI:** 10.3389/fpls.2021.673576

**Published:** 2021-10-18

**Authors:** Rahul B. Nitnavare, Joorie Bhattacharya, Satnam Singh, Amardeep Kour, Malcolm J. Hawkesford, Naveen Arora

**Affiliations:** ^1^Division of Plant and Crop Sciences, School of Biosciences, University of Nottingham, Nottingham, United Kingdom; ^2^Department of Plant Science, Rothamsted Research, Harpenden, United Kingdom; ^3^International Crops Research Institute for the Semi-Arid Tropics (ICRISAT), Hyderabad, India; ^4^Department of Genetics, Osmania University, Hyderabad, India; ^5^Punjab Agricultural University (PAU), Regional Research Station, Faridkot, India; ^6^Department of Biointeractions and Crop Protection, Rothamsted Research, Harpenden, United Kingdom; ^7^Punjab Agricultural University (PAU), Regional Research Station, Bathinda, India; ^8^Department of Genetics and Plant Breeding, Punjab Agricultural University (PAU), Ludhiana, India

**Keywords:** RNAi silencing, dsRNA, siRNA, coleopteran, hemipteran, lepidopteran, nanoparticles, transgenics

## Abstract

RNA interference (RNAi) is a method of gene silencing where dsRNA is digested into small interfering RNA (siRNA) in the presence of enzymes. These siRNAs then target homologous mRNA sequences aided by the RNA-induced silencing complex (RISC). The mechanism of dsRNA uptake has been well studied and established across many living organisms including insects. In insects, RNAi is a novel and potential tool to develop future pest management means targeting various classes of insects including dipterans, coleopterans, hemipterans, lepidopterans, hymenopterans and isopterans. However, the extent of RNAi in individual class varies due to underlying mechanisms. The present review focuses on three major insect classes *viz* hemipterans, lepidopterans and coleopterans and the rationale behind this lies in the fact that studies pertaining to RNAi has been extensively performed in these groups. Additionally, these classes harbour major agriculturally important pest species which require due attention. Interestingly, all the three classes exhibit varying levels of RNAi efficiencies with the coleopterans exhibiting maximum response, while hemipterans are relatively inefficient. Lepidopterans on the other hand, show minimum response to RNAi. This has been attributed to many facts and few important being endosomal escape, high activity dsRNA-specific nucleases, and highly alkaline gut environment which renders the dsRNA unstable. Various methods have been established to ensure safe delivery of dsRNA into the biological system of the insect. The most common method for dsRNA administration is supplementing the diet of insects *via* spraying onto leaves and other commonly eaten parts of the plant. This method is environment-friendly and superior to the hazardous effects of pesticides. Another method involves submergence of root systems in dsRNA solutions and subsequent uptake by the phloem. Additionally, more recent techniques are nanoparticle- and *Agrobacterium*-mediated delivery systems. However, due to the novelty of these biotechnological methods and recalcitrant nature of certain crops, further optimization is required. This review emphasizes on RNAi developments in agriculturally important insect species and the major hurdles for efficient RNAi in these groups. The review also discusses in detail the development of new techniques to enhance RNAi efficiency using liposomes and nanoparticles, transplastomics, microbial-mediated delivery and chemical methods.

## Introduction

The first reported RNA interference (RNAi) was in the nematode, *Caenorhabditis elegans* by Fire et al. (1998) wherein mRNA silencing was observed with exogenous dsRNA. Thereafter, RNAi has been explored in various plants, insect orders such as Coleoptera (Palli, 2014), Lepidoptera (Kolliopoulou and Swevers, 2014), Hemiptera (Li et al., 2013b; Singh et al., 2019a), Diptera (Maktura et al., 2021), Thysanoptera (Singh et al., 2019b) and animal systems (Martin and Caplen, 2007). RNA interference is the mechanism of dsRNA-mediated gene silencing by the digestion of dsRNA by DICER enzyme into small interfering RNAs (siRNA). These siRNAs then are incorporated into the RNA-induced silencing complex (RISC). The RNAi are homologous to the target mRNA and hinder further translation in the cell. The uptake mechanism of RNAi in insects can mainly be divided into two types, *viz.*, cell-autonomous and non-cell autonomous RNAi. In case of cell-autonomous, the silencing is seen only in the tissues wherein the dsRNA has been introduced rendering limited efficiency. On the other hand, in case of non-cell-autonomous RNAi, the effect of silencing is also observed in locations other than the site of application of dsRNA. In insects, non-cell-autonomous RNAi has been widely explored wherein the internalisation of dsRNA is done through direct feeding. The silencing is achieved when the dsRNA is taken up into the gut cells from the gut lumen. Thereafter, the dsRNA is expressed in cells outside of the gut which allows for spread of the silencing signal (Huvenne and Smagghe, 2010).

The uptake of dsRNA has been observed through various mechanisms. Several transmembrane proteins facilitate the uptake of dsRNA in the gut of insects. SID-1 and SID-2 have been known to coordinate the transport of dsRNA (Winston et al., 2007). In *C. elegans* SID-2 plays a role in uptake of environmental dsRNA whereas SID-1 is a key component for systemic RNAi (Winston et al., 2002). SID- 2 has no role in the spread of RNAi among the cells. The expression of SID-1 in *Drosophila* S2 cells, which lack the *SID-1* gene resulted in high RNAi response even at low dsRNA concentrations (Shih et al., 2009). However, in *Drosophila melanogaster* an autonomous mode of silencing implied the uptake of dsRNA through endocytosis wherein, it would be directly delivered to the cytoplasm of the cell. Vacuolar H^+^ ATPase have been found to be associated with this dsRNA uptake system. This mechanism has been found to be evolutionarily conserved as it was seen to occur in other organisms, namely, *C. elegans* (Saleh et al., 2006). SID-1 like proteins have been reported from most of the insect species; however, so far SID-2 like proteins seem to be absent in insects (Tomoyasu et al., 2008; Cappelle et al., 2016) Furthermore, the innate immune system also was found to play a role in RNAi. Infected cells were found to release dsRNA which was taken up by the infected cells *via* the cellular dsRNA uptake pathway. This involved autonomous as well as a systemic immunity imparted to the infected cell to protect it from further viral spread. This mechanism has been found to be facilitated by specific scavenger receptors (SR) such as Sr-CI, Sr-CII, Sr-CIII, Sr-CIV, croquemort and epithelial membrane protein. These SR-like genes have been identified in *D. melanogaster* (Saleh et al., 2009). The various mechanisms involved in RNAi have been explored in a wide range of insect classes/orders such as Coleoptera, Diptera, Hemiptera, Hymenoptera, Lepidoptera, Orthoptera and Isoptera and studied for extent of mRNA silencing capacity (Huvenne and Smagghe, 2010; Singh et al., 2017).

Direct ingestion of dsRNA has been effectively used for the purpose of RNAi over the years and exhibits potential for pest management due to its specificity as well as ease of introduction into insect cell. dsRNA can be introduced into insect systems by spraying onto infested crops and enhancing specificity. This technique has been seen as an alternative to usage of pesticide and has gained popularity as it also poses reduced damage to the environment. Additionally, silencing through dsRNA exhibits modest risk to development of resistance. In spite of these advantages, researchers have come across several shortcomings in the process of pest management using RNAi such as degradation of the dsRNA before ingestion, variability in specificity within species and lack of efficient trans-membrane protein channel for uptake of the dsRNA. Degradation of dsRNA has been observed post-ingestion due to the presence of digestive nucleases and the inherent gut pH of the insect. Numerous technologies have been adopted and implemented to overcome these challenges. Various techniques and pathways for the effective oral administration of the dsRNA into the gut of the insect have been explored (Kourti et al., 2017, Kunte et al., 2020). In an experiment in which the root system of tomato plant was immersed in a solution containing dsRNA, an astounding 80% mortality rate of the tomato pest, *Tuta absoluta*, was observed implying an efficient uptake of the dsRNA by the phloem system (Majidiani et al., 2019). Another technology which has been studied is the usage of nanoparticles as molecular carriers of dsRNA across insect gut epithelia. Nanoparticles are known to be able to protect the dsRNA from nucleases. Several nanoparticle systems have been used for this purpose including liposomes, chitosans and branched amphiphilic peptide capsules (BAPCs) in insect classes such as Lepidoptera, Coleopterans, Diptera and Dictyoptera. Due to the ability to overcome cellular and extracellular barriers for dsRNA delivery, nanotechnology has been widely adopted. Since it is a recent technology, researchers are still working on perfecting this technique due to issues such as ability of the nanoparticle to unbind the dsRNA and allow the appropriate enzyme to process it. To ensure this, the ratio of the nanoparticle and dsRNA must be suitably selected. Additionally, there have been reports of nanoparticles being cytotoxic beyond a threshold concentrations (Tripathi et al., 2017). Another delivery mechanism is feeding insects with transgenic plants developed *via Agrobacterium*-mediated transformation. Many studies have used this technique to develop resistance against hemipterans, lepidopterans and coleopterans and is named as the nuclear transformation method. In utilization of the plants’ inherent RNAi machinery for generation of dsRNA, it is often processed into siRNA before ingestion by the insect species. This causes a hindrance in the development of dsRNA-transgenic plants. Therefore, in order to avoid this, the expression of dsRNA is done in organelles such as, chloroplasts where the dsRNA is protected from degradation by plants own RNAi machinery. Nuclear dsRNA localizing plants obtained through nuclear transformation also exhibit no insect mortality rate unlike its chloroplast counterpart which showed up to 100% mortality in certain insect species. However, due to recalcitrant nature of several crops and lack of regeneration protocols, chloroplast dsRNA-expressing delivery mechanism has not yet been expanded (Kunte et al., 2020). In an extension to this concept, engineered microorganisms such as bacteria and yeast containing introgressed dsRNA have been used to deliver dsRNA into the insect gut by orally delivering the microorganisms. This technique has been implemented using both live and heat-killed microorganisms. Live microbes were seen to be comparatively more potent (Tian et al., 2009). Delivery using viruses, although potentially effective, has yet to be considered as a compelling strategy due to the inherent virus-induced silencing effects. This method faces issues in developing appropriate symbiotic organisms, the transformation method and re-colonization (Kunte et al., 2020).

A wide array of such methods are being studied for sustainable oral delivery of dsRNA into insect system taking into consideration regulatory as well as environmental aspects. One of the problems faced by RNAi is the off-target effect of siRNAs after being processed from the dsRNA. siRNAs are usually small sequences and thus often bind with non-target genes which might cause deleterious effects. This may pose a serious problem for non-target organisms (NTO). In this case, the designing of dsRNA is crucial, whereby even a single base pair mismatch would prevent binding to off-targets (Scott et al., 2019). Bioinformatics tools are being developed which provide a systemic evaluation of RNAi off-target effects which can be supported by assays to validate them. These tools enable the generation of dsRNAs which are unique and thereby minimize off-targets. These tools should additionally consider any potential interaction within a pool of siRNAs. Generation of longer siRNAs may be a noteworthy alternative as they would be less prone to off-targets (Hannus et al., 2014). Additionally, it is vital to consider the role of the other factors for effective dsRNA delivery such as nature of the gene targeted, specificity and length of the dsRNA. Significant challenges need to be addressed for individual techniques to have conclusive and effective pest control in the field environment. These aspects of RNAi are elaborated below.

**Figure 1:**
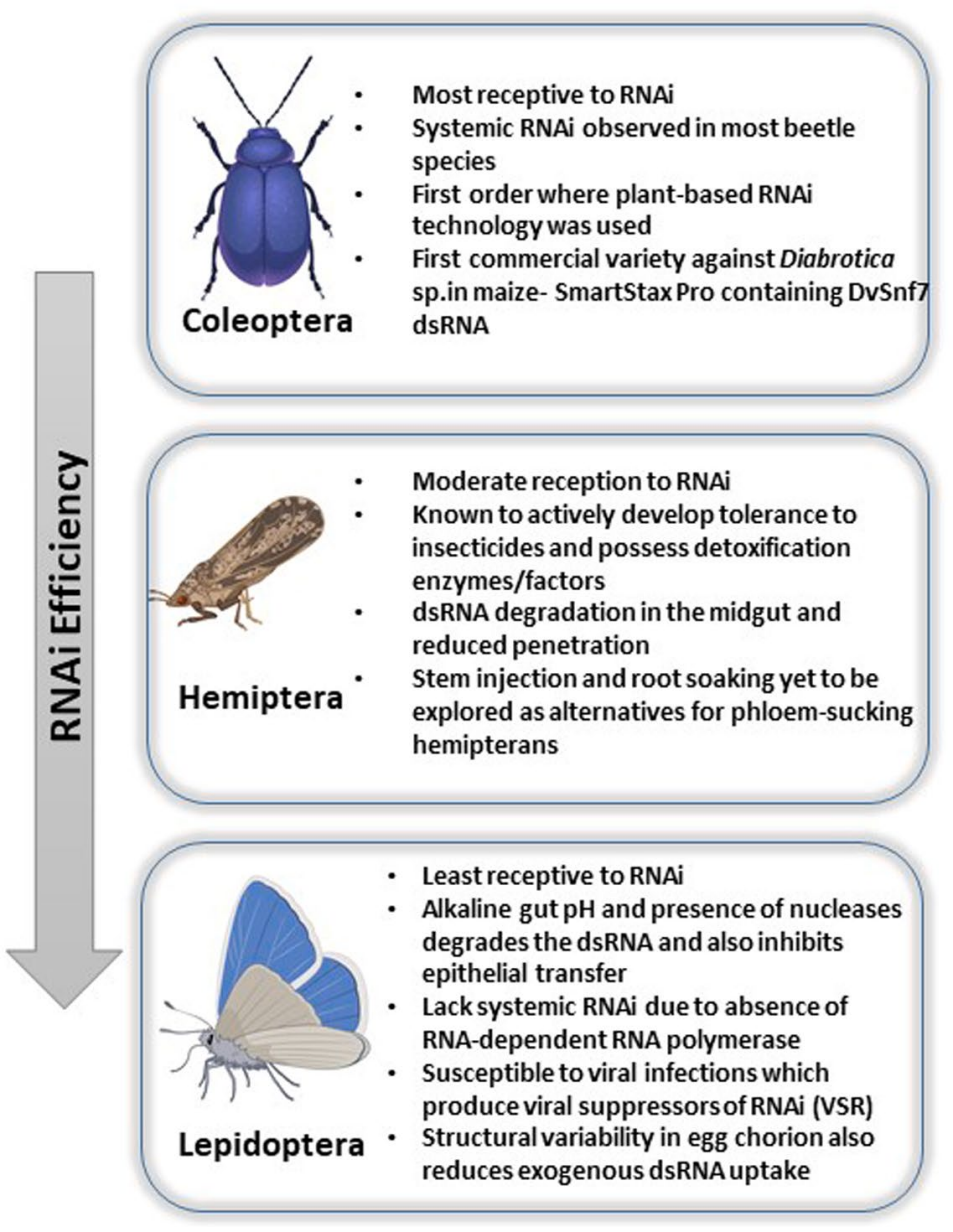
Pictorial representation of RNAi efficiencies and challenges faced across insect orders like Coleoptera, Hemiptera, and Lepidoptera.

Interestingly, the silencing efficiency varies amongst the insect classes where Coleopterans often exhibit 100% susceptibility and hemipterans, on the other hand, exhibit reduced efficiency. Lepidopterans, on the other hand, are the most recalcitrant to oral RNAi owing to their highly alkaline gut environment. Thus, the three orders focused in this review show diverse response to RNAi and include most of the insects of agricultural importance where RNAi has been exhaustively studied. The variability in efficiency has been attributed to several factors such as variability in gut nuclease, pH, sucking/chewing mouthparts as well as length of the dsRNA molecule (Majidiani et al., 2019). The following review expands on this and explains the various mechanisms of delivery in three important classes of insects, namely, coleopterans, hemipterans, and lepidopterans (Figure 1), wherein RNAi studies have been extensively carried out along with analysis of the persistency and the underlying mechanisms.

## Rnai in Coleopterans

The order Coleoptera has been known to be most conducive to the RNAi technology (Shukla et al., 2016). This is also the first known order to be targeted using transgenic technology *via* RNAi plants (Rodrigues and Figueira, 2016; Table 1.).

**Table 1:** A comprehensive table depicting the various genes targeted across major Lepidoptera, Coleoptera, and Hemiptera species for pest control using RNAi technologies.

Insect order	Target insect	Target gene/trait	References
Lepidoptera	*Bombyx mori*	Homeodomain transcription factors	Deng et al., 2012
	*Choristoneura fumiferana*	Chitin deacetylase	Quan et al., 2013
	*Helicoverpa armigera*	Cytochrome P450 enzyme system	Tang et al., 2012
	*Helicoverpa armigera*	HMG-CoA reductase	Wang et al., 2013
	*Sesamia nonagrioides*	Juvenile hormone esterase related gene	Kontogiannatos et al., 2013
	*Spodoptera exigua*	Nine genes, including tubulin, chitinase, ARF1, carboxypeptidase, ATPase, helicase	Li et al., 2013a
	*Spodoptera littoralis*	Period clock gene	Kotwica-Rolinska et al., 2013
	*S. exigua*	chitin synthase gene A (SeCHSA)	Tian et al., 2009
	*B. mori*	Pigmentation causing gene	Quan et al., 2002
	*Hyalophora cecropia*	Hemolin	Bettencourt et al., 2002
	*Spodoptera litura*	*Bt* toxin receptor	Rajagopal et al., 2002
	*H. virescens*	Role of intracellular transportation and unstable dsRNA in RNAi	Shukla et al., 2016
	*Spodoptera frugiperda*	Polymer coated dsRNA	Parsons et al., 2018
	*Spodoptera exigua*	Guanidine coated polymer for dsRNA against chitin synthase	Christiaens et al., 2018
	*Helicoverpa zea*	Gene knockout studies and dsRNA against neuronal channels	Wang et al., 2018a
	*Bombyx mori*	Overexpression of *Ago2* and *Dicer*2 proteins	Li et al., 2015b; You et al., 2020
Hemiptera	*Bemisia tabaci*	Cytochrome P450 monooxygenases associated with resistance	Li et al., 2015b
	*Nilaparvata lugens*	NADPH–cytochrome P450 reductase	Liu et al., 2015
	*Laodelphax striatellus*	Cytochrome P450 CYP4DE1 and CYP6CW3v2	Elzaki et al., 2015
	*Laodelphax striatellus*	Cytochrome P450 monooxygenase	Wan et al., 2015
	*Bemisia tabaci (Genn.)*	Acetylcholinesterase (*AChE*) and ecdysone receptor (*EcR*) homologous genes of *B. tabaci*	Malik et al., 2016
	*Aphis gossypii*	Juvenile hormone-binding protein (*JHBP*) and vacuolar ATPase subunit H (*V-ATPase-H*)	Rebijith et al., 2016
	*Bemisia tabaci*	Inositol 1,4,5-trisphosphate	Guo et al., 2017
	*Diaphorina citri*	Acetylcholinesterase	Kishk et al., 2017
	*Aphis(Toxoptera) citricidus* (Kirkaldy)	Acetylcholinesterase	Mou et al., 2017
	*Bemisia tabaci*	Hydroxyacidoxoacid transhydrogenase	Yang et al., 2017
	*Sitobion avenae*	*Laccase 1*	Zhang et al., 2018
	*Bemisia tabaci*	Glutathione S-transferase (GST), BtGSTs5	Eakteiman et al., 2018
	*Sitobion avenae F.*	Olfactory-related Gq𝜶 gene	Hou et al., 2019
	*Bemisia tabaci*	ABC transporter genes	He et al., 2019
	*Diaphorina citri*	UDP-Glycosyltransferases genes	Tian et al., 2019
	*Bemisia tabaci*	RNAi silencing using piwi-associated RNAs (piRNA)	Mondal et al., 2020
Coleoptera	*Diabrotica* spp.	Silencing the DvSnf7 gene which is involved in intracellular trafficking	Bolognesi et al., 2012
	*Diabrotica virgifera*	Targeting the ATPase gene which is a housekeeping gene	Rangasamy and Siegfried, 2012
	*Diabrotica virgifera*	Targeting two chromatin remodelling genes which affects the sperm viability using pRNAi.	Vélez et al., 2017
	*Tribolium* sp.	Leg gene, a *Tribolium* homologue of *proboscipedia* (*pb*) and homeotic genes using pRNAi	Bucher et al., 2002
	*Leptinotarsa decemlineata*	*β-actin* and *shrub* proteins expressing transgenic potato in the chloroplast	Rodrigues and Figueira, 2016
	*Leptinotarsa decemlineata*	Bacterial-mediated dsRNA delivery using *E. coli* deficient in RNaseIII enzyme which degrades dsRNA	Palli, 2014
	*Plagiodera versicolora*	*E. coli* deficient in RNaseIII was fed containing dsRNA targeting *ACT*, *SHI*, *CACT*, *SRP54*, *HSC70*, and *SNAP*	Zhang et al., 2019
	*Cylas puncticollis*	dsRNA against sclerotization gene led to death of larva	Prentice et al., 2015
	*Leptinotarsa decemlineata* and *Tribolium castaneum*	Studies on *StaufenC* responsible for enhanced RNAi in coleopterans	Yoon et al., 2018

It has been observed that in most beetles, even small quantities of dsRNA are sufficient to induce the RNAi machinery in both the larval and adult stages. Systemic RNAi has also been seen in majority of beetle species while variable RNAi has also been known, which is most likely due to the oral delivery of dsRNA. In the study by Baum et al. (2007), significant strides were made in defining the parameters required for efficient RNAi. Western corn rootworm, WCR (*Diabrotica virgifera virgifera*), is an economically important agricultural pest and was found to be highly receptive to RNAi silencing, irrespective of the delivery method used. WCR, which feeds on the roots of maize crops, saw significant mortality and stunting in larva expressing a hairpin version of a housekeeping ATPase gene. The Snf7 ortholog, which encodes an essential intracellular trafficking protein and was seen to cause WCR mortality was selected as target mRNA in WCR. The WCR Snf7 (DvSnf7) is a part of the endosomal sorting complex and plays a role in sorting of cell membrane receptors. When the protein targeting dsRNA was fed to adult WCR, inhibition of growth and subsequent death was observed. Expression studies using real-time PCR demonstrated that insects which were fed with 60ng/ml of DvSnf7 dsRNA for 5days show mRNA level suppression as compared to insects fed with control diets. Additionally, in order to assess the DvSnf7 protein levels in the insects, anti-Snf77 antibody was synthesized and the larval samples were examined through western blot and ELISA. After 5days of feeding, the protein levels were seen to be reduced as compared to the 1st day of exposure. The diet bioassay data along with molecular studies successfully exhibited the applicability of dsRNA in causing effective mortality of WCR (Bolognesi et al., 2012, Rangasamy and Siegfried, 2012). Another type of RNAi mechanism which exists is Parental RNAi (pRNAi). In this, the effect of the targeted gene is seen in the progeny instead of the targeted organism. The genes usually targeted are the embryonic development-based genes which lead to a reduction in hatched eggs or a lack of viable larvae, whilst the adult remains unaffected. This mechanism was exhibited in WCR, in which two chromatin remodelling genes were targeted and studied for effect on sperm viability and fecundity. A distinctive effect was seen on females, while subtle effects were seen on sperm count (Vélez et al., 2017). This has also been studied in *Tribolium* sp. in which three genes, leg gene, a *Tribolium* homologue of *proboscipedia* (*pb*) and homeotic genes were targeted in the species. An RNAi phenotype was observed in 100% of the progeny in the first generation and it may be concluded to be highly effective for coleopterans. However, this number was seen to gradually decrease in the subsequent generation, which may be due to degradation of dsRNA and consequential loss of activity (Bucher et al., 2002). These studies formed the basis for plant-based RNAi at field level for longer effectivity of RNAi extended even up to the next generation (Alamalakala et al., 2018).

Another important pest belonging to Coleoptera order is the Colorado potato beetle, CPB (*Leptinotarsa decemlineata*). Induction of RNAi machinery by expressing dsRNA in the chloroplast and not in the nucleus has been well exploited due to the absence of dicer protein in the former. This allows the dsRNA to remain intact unlike in the nucleus. Transgenic potato plants which expressed *β-actin* and *shrub* dsRNA in the chloroplast were able to impart pesticidal activity in beetle whereas in nuclear expressed dsRNA, no RNAi was seen into effect (Rodrigues and Figueira, 2016). Then bacterial-mediated dsRNA delivery mechanism has also proven to be effective in CPB. *Escherichia coli* deficient in RNaseIII enzyme, which degrades dsRNA, was used to produce dsRNA. The dsRNA which was developed to target housekeeping genes in CPB demonstrated larval mortality and reduced insect growth due to decrease in feeding and as a consequence of loss of function of the genes. Several other genes encoding for essential proteins such as, actin, the ryanodine receptor, Shade-a gene coding for P450 enzyme and S-adenosyl-L-homocysteine, have been targeted in CPB using bacteria producing dsRNA and showed significant effects on the insect mortality and growth implying towards the robustness of this technique in coleopterans (Palli, 2014). With the advent of genetic engineering, transgenic plants expressing *Bacillus thuringiensis* toxins such as the *Cry* proteins, have been developed. Over many years of use of pest management strategies, CPB has developed resistance to insecticides and *Bt* toxin. There is a possibility that tolerance to RNAi could develop; however, there is no such recorded instance of resistance to RNAi in coleopterans to date. RNAi has been exploited for CPB control and several delivery methods and multiple target genes have been identified in this insect order. Genes related to growth, mortality and flight competence in adults, ability of moulting and pupation in larva, and enhancement of *Bt* toxicity have been extensively targeted and studied in CPB. Studies pertaining to the gut of coleopterans have confirmed the presence of all the RNAi-related genes, which contribute to its acceptance to RNAi silencing. These genes contribute to essential cellular pathways and detoxification processes and whose suppression or knockout can directly induce mortality. Thus, they can be targeted for CPB control. Exploring these genes might help in understanding the exact RNAi pathway in CPB and its comparative enhancement to RNAi as compared with other species and orders of insects (Mei-qi et al., 2020).

Bacterial-mediated dsRNA delivery methods have been exploited in several species as they prove to be an excellent alternative for pest control methods. Apart from CPB, this method has been used to reduce the harmful effects of *Plagiodera versicolora*, which is an important pest of the Salicaceae family of plants, causing serious infestations worldwide. RNaseIII-deficient *E. coli* which contained dsRNA targeting six vital genes, *viz.*, *ACT*, *SHI*, *CACT*, *SRP54*, *HSC70*, and *SNAP*, was fed to this beetle. Most of these genes were found to be silenced in *P. versicolora* while, ACT and SRP54 caused significant percentage of mortality. A more recent plastid-based expression of dsRNA has been developed in poplar species which has shown results for certain Lepidopterans. This work can be extrapolated to other orders including Coleoptera, which are more receptive to dsRNA. Transplastomic expressing dsRNA can be targeted to *β-actin* and *shrub* proteins to study its effect on *P. versicolora*. Bacterial means of dsRNA delivery is scalable and cost-effective as bulk production of dsRNA is more expensive as compared to this technology. They can be easily sprayed upon plants and causes no detrimental effects to the environment. However, there are still a few aspects of this technique which need to be taken into consideration before full-scale application including potential of resistance development, off-targets on gene as well as organism and mutation of RNAi components (Zhang et al., 2019). In order to overcome the issue of development of resistance, symbiont bacteria are being explored for constitutive and a trauma-free delivery of dsRNA into the insect system (Whitten et al., 2015).

RNAi technology has shown potential in other important coleopteran species such as African sweet potato weevil, SPW (*Cylas puncticollis*). A strong systemic RNAi pathway was found to be prevalent in SPW when dsRNA targeting a gene involved in sclerotization of the insect exoskeleton was injected into the second instar larvae. Sclerotization was inhibited leading to eventual death of the larva (Prentice et al., 2015). Delivery of dsRNA orally and by injection was also carried out targeting the *Snf7* gene in SPW, which is usually targeted in WCR. Microinjection is not a realistic method of RNAi application under field conditions; however, it is an efficient tool for initial evaluation/validation of dsRNA mediated knockdown under laboratory conditions. Microinjection showed promising results and comparable results with the effect of RNAi in WCR whilst the oral delivery method was studied for application in the field. For this, a total of three genes were targeted which were involved in various translational and transport pathways. In this method, degradation of dsRNA was observed which was concluded to be due to the effect of gut nucleases. These results were relative to similar studies carried out in certain hemipterans and lepidopterans. Supplementing this with artificial diet however, prolonged the stability of the dsRNA in the gut. Therefore, strategies to enhance the stability of the dsRNA in the insect should be developed which would protect it from nucleases. One such way is continuous exposure of the dsRNA by frequent dosage supplementation to the insect. The other alternative to establish consistent delivery to the insect is *via* transgenic plants (Prentice et al., 2017).

While RNAi technology is found to be quite effective in coleopterans, it still poses the risk of exposure to NTO, which can be natural enemies to various pests as well. Before approval of field trials and commercialization of RNAi silenced plants, an environmental risk assessment (ERA) is necessary which considers the potential risks to the ecosystem. The ERA is carried out by assessing the effect of RNAi on a wide range of insects which are of value to the ecosystem. MON 87411 maize expressing *DvSnf7* dsRNA for protection against *Diabrotica* sp. was assessed against potential NTO. However, the study showed that *DvSnf7* has a narrow activity spectrum and is specific to only a certain sub-family of beetles. No detrimental effects were observed in any of the tested NTOs and hence *DvSnf7* dsRNA was concluded to be safe for field trials (Bachman et al., 2013, 2016). Ladybird beetles are often taken up for such studies as they are predators of pests and are at a risk of such technologies. Apart from feeding on smaller insects and pests, they are known to also consume plant parts which puts them at a risk in case they consume genetically engineered plants. *Adalia bipunctata* and *Coccinella septempunctata*, two ladybird species are important natural predators. Their susceptibility to RNAi was studied upon exposure to dsRNA targeted against ATPaseA of *Diabrotica virgifera*. It was seen that the two ladybird species are sensitive to dsRNA but differed amongst themselves. Also, it was seen that the exposure to dsRNA was damaging at concentrations above threshold levels usually present in the field which is the worst case scenario (Haller et al., 2019). Further studies to enunciate these results would help give a comprehensive picture of the actual effects of dsRNA on natural enemies.

The cause of the evident variation which occurs in the various orders and the high sensitivity of coleopterans to RNAi is not well known. A dsRNA binding protein, StaufenC, was identified which was determined to play a vital role in RNAi. This protein has been found to exist only in coleopteran order and functions in the processing of dsRNA to siRNA and was studied in *L. decemlineata* and *Tribolium castaneum*. This suggested a correlation between presence of *StaufenC* and robust systemic RNAi in coleopterans. This study was extremely significant as it was able to provide some insight into the mechanism of RNAi and the variation which occurs between different orders (Yoon et al., 2018).

As explained above, Coleopterans are highly receptive to RNAi technology and have been widely studied in different organisms taking into consideration techniques of delivery as well as newer techniques such as transplastomic RNAi generation. There is however, still further scope to increase the durability and of dsRNA and managing resistance development in the different species.

## Rnai in Hemipterans

RNAi has been carried out in various hemipteran species such as aphids and planthoppers. The technology is moderately effective in hemipterans (Table 1); however, there are few challenges which the order poses. It has been observed that RNAi is effective when targeted against genes of the midgut and salivary glands. However, when insects are fed with transgenic plants expressing dsRNA, the technology has shown to be less efficient. Three major methods of delivery have been adopted for dsRNA in hemipterans, *viz.*, microinjection, oral delivery and feeding of transgenic plant. Microinjection is the least used method amongst of all of these as it is comparatively more expensive and provided no room for manipulation due to immune response trigger by physical damage caused to the minute insect. Feeding an oral diet containing dsRNA has shown to be effective in hemipterans (Singh et al., 2018). It is an easier way of dsRNA delivery and causes no damage to the insect. Moreover, unlike microinjection which is done at an adult stage, oral feeding can be done even at early nymph and larval stages. However, this method still lacks the ability to produce dsRNA in excess amount along with having a low silencing efficiency. Furthermore, another technique which has been implemented in hemipterans is the generation of transgenic plants producing dsRNA targeting specific insect vital genes. The major idea to keep in mind for this technique is the expression of sufficient amounts of dsRNA able to produce lethal phenotypes in the insects. RNAi is effected by numerous factors in hemipterans such as the mode of delivery, the concentration of dsRNA and also the quality and design of dsRNA. The dsRNA designed should be such that it is highly specific to the target gene and unique in its binding ability. This step is extremely crucial as it should be ensured that the possibility of non-specific targeting of other genes and organisms is negated. Additionally, the size of the dsRNA is another aspect as most of the conclusive research has used dsRNA ranging from 185 and 675bp. Usually, siRNAs are known to produce off-targets (Li et al., 2013a). Different species of hemipterans which have been studied to further explore this technology are discussed below.

The *Bemisia tabaci*, or the tobacco whitefly, is one of the most hazardous pests effecting vegetable and ornamental crops. Cytochrome P450 monooxygenases are associated with pesticide resistance in *B. tabaci*. dsRNA specific to the P450 CYP6CM1 gene of the insect species was synthesized and introduced into the insect *via* membrane feeding. Two biotypes of *B. tabaci* were taken for this purpose and it was seen that silencing occurred in both the biotypes within a range of 55–85%. High mortality was observed in the biotypes as well as inability to detoxify specific pesticides (Li et al., 2015a). On similar lines, the role of NADPH-cytochrome P450 reductase (CPR) has been studied in *Nilaparvata lugens*, with regard to the tolerance to the insecticides, beta-cypermethrin and imidacloprid. The study provided the first evidence for susceptibility to insecticides upon down regulation of CPR in *N. lugens* (Liu et al., 2015). CPR Targeting dsRNA specific to CPR demonstrated susceptibility to the two insecticides as compared to the control insect. Whitefly has also developed resistance to another major insecticide, anthranilic diamide, wherein cyantraniliprole is the second active ingredient. In this, the inositol 1,4,5-trisphosphate receptor (IP3R) is activated upon exposure to the pesticide. The receptor is encoded by the gene, *BtIP3R*. dsRNA targeting this gene has shown significant reduction in tolerance to cyantraniliprole in *B. tabaci*. This study can be further explored for studying the resistance mechanism of similar species against cyantraniliprole and corresponding insecticide resistance (Guo et al., 2017). Another factor contributing to detoxification mechanisms of insecticides is the ABC transporter. In various studies an increase in the expression of ABC transporters has been observed upon exposure to insecticides. For this purpose, *B. tabaci* has been exposed to imidacloprid insecticide and expression studies revealed that the ABCG sub-family showed increased expression. RNAi knockdown was performed on the gene sub-family and a noticeable mortality rate increase was observed in imidacloprid exposed *B. tabaci*, implying an ability of the ABC transporter to detoxify the insecticide (He et al., 2019). *Bemisia tabaci* has also developed a high resistance to thiamethoxam insecticide, and the gene hydroxyacidoxoacid transhydrogenase (*HOT*) has been studied to impart tolerance. Knockdown of the *HOT* gene showed a drastic reduction in thiamethoxam resistance upon feeding the insect a diet containing *HOT* dsRNA (Yang et al., 2017). Since hemipterans are usually phloem-feeding and contain sucking mouthparts, plant-mediated RNAi delivery is the most efficient in the species. Among the many insecticide resistance genes identified in hemipterans, most of them are triggered due to extensive exposure to uncontrolled use of insecticides. In *B. tabaci*, a glutathione S-transferase (GST) gene, *BtGSTs5* was targeted using RNAi by expressing the *BtGSTs5* dsRNA in *Arabidopsis*. Silencing of the gene also showed the extension of the nymph developmental period. The study implied that targeting of the GST gene may not altogether lead to death of the pest, but will allow the effect of insecticides as well as will provide time for activity of natural enemies to take over (Eakteiman et al., 2018). *Acetylcholinesterase* (*AChE*) and *ecdysone receptor* (*EcR*) are vital genes which are involved in major developmental processes of insects. Plant mediated dsRNA delivery to *B. tabaci* was performed by developing transgenic *Nicotiana tabacum* L. expressing dsRNA targeting *AChE* and *EcR*. Downregulation of these genes led to increased mortality of *B. tabaci* for upto 90% as compared to non-dsRNA expressing plants fed insects. Additionally, *B. tabaci* is also a vector for plant viruses such as *Tobacco rattle virus* and therefore, using RNAi, the spread of plant viruses can also be reduced (Malik et al., 2016). The potential of transgenic plant-based RNAi silencing needs to be explored further for phloem-feeding insects by identifying appropriate receptors and optimizing strategies for the same. Apart from miRNAs and siRNAs, another class of small RNAs are the piwi-associated RNAs (piRNAs). These are normally restricted to the germline of other insect species but are found abundantly in the soma of hemipterans. The piRNAs are *dicer* independent and are mediated by three proteins, *viz.*, PIWI, aubergine and argonaute 3. While the presence of piRNA pathways has been studied in several insects including hemipterans, using piRNAs as a novel strategy for RNAi silencing has yet to be extensively investigated (Brito et al., 2018). Studies performed on *B. tabaci* exhibited a potential rationale for piRNA-based gene silencing. The study also suggested an effect of piRNA upon exogenous delivery and subsequent introduction into the piRNA pathway. Compared to coleopterans, hemipterans have only a moderate response to RNAi due to the degradation of dsRNA in the midgut as well as reduced penetration capability. This requires a high dosage of dsRNA to compensate for the degradation. Therefore, the prospect of piRNA mediated silencing is quite promising. More so because the piRNA triggers to RNAi are comparable with siRNA triggers. Additionally, this could be an alternative approach to insect species which are recalcitrant to dsRNA (Mondal et al., 2020).

The wide research done on *B. tabaci* is due to the availability of the genome database. Apart from genes governing ecdysis, moulting signalling, reproduction, and various insecticide receptors, genes associated with sugar metabolism, transport, have also been known to be vital for whitefly viability and have been studied using RNAi. It has been evident that plant-based RNAi delivery produces higher levels of dsRNA and consequently a higher mortality rate for whitefly. However, root and stem absorption have not been explored yet for whiteflies which are excellent methods for phloem-feeding insects. Another method which has been explored is the use of endosymbionts which reside in the gut of the insect. As introduction of other microorganisms may trigger an immune response, scientists have used previously residing gut microflora to achieve RNAi silencing. Such a study has been carried out in *Rhodnius prolixus*, wherein a dsRNA cassette was introduced into the gut endosymbiont of the insect (Whitten et al., 2015). In *B. tabaci*, several such primary and secondary symbionts exist which can be used for this purpose. Further studies on the usage of endosymbionts can open an entirely new dimension for RNAi studies in hemipterans (Grover et al., 2019).

Similarly to whitefly, the melon or cotton aphid (*Aphis gossypii*) is a major pest globally, infecting crops and acting as a vector for viral diseases. The aphid has a complex life cycle and has developed resistance to various insecticides causing a serious problem worldwide. Diet-mediated delivery of dsRNA against the vacuolar ATPase subunit H (V-ATPase-H) and juvenile hormone-binding protein (JHBP) has resulted in mortality of the aphid ranging from 10 to 63% for both the genes (Rebijith et al., 2016). Acetylcholinesterase (*AChE*) is the primary gene targeted by the insecticides malathion and carbaryl in the aphid, *Aphis (Toxoptera) citricidus* (Kirkaldy). Malathion and carbaryl are organophosphates and carbamates-based insecticides. RNAi silencing of two *AChE* genes of the aphid, *Tcace1* and *Tcace2* encoding TcAChE1 and TcAChE2, showed increased susceptibility to the insecticides. However, silencing of *Tcace1* resulted in higher mortality in the aphid as compared to *Tcace2* due to its role in neurotransmission (Mou et al., 2017). Among the aphids studied, the grain aphid (*Sitobion avenae*) is an important pest of cereals and damages the crop by sucking on the sap of the plant. Targeting genes which are directly involved in metabolic and signal transduction pathways using gene silencing has proven to be effective in reducing the aphid population. G-proteins, which form a part of the signal transduction pathway, play a role in olfaction, sight and signalling of the endocrine system. A sub-family of G-proteins called *Gq* proteins mediate olfactory signals and exist as trimer sub-units, *viz.*, alpha (α), beta (β) and gamma (ɣ). Transgenic wheat expressing *Gqα* dsRNA was fed to *S. avenae* which substantially reduced the level of *Gqα* in the aphid resulting in reduced moulting and reproduction. The experiment was carried out in field conditions and not only showed reduced aphid numbers but also increased grain weight as compared to the control (Hou et al., 2019). Plant mediated RNAi has also been carried out to target the *laccase1 (Lac1)* gene in *S. avenae*. *Lac1* is involved in immune response and iron metabolism of the aphid and is expressed abundantly in the mid gut and salivary gland. Knockdown of the *Lac1* by targeting through orally fed dsRNA reduced the survival rate of *S. avenae*. It was also found that the gene had potential to detoxify toxic phenolic secondary metabolites and eventual plant susceptibility (Zhang et al., 2018).

The small brown planthopper (*Laodelphax striatellus*) is an economically important pest of rice. Repeated insecticide exposure due to implementation of management strategies has led to the pest developing insecticide resistance. The gene cytochrome P450 monooxygenase Shadow (*Sad*) plays a role in ecdysis. dsRNA targeting the *Sad* gene of *L. striatellus* by oral delivery reduced the expression of the ecdysone receptor and caused prolonged nymphal development and eventual death (Wan et al., 2015). *Laodelphax striatellus* shows resistance to ethiprole insecticide. Two cytochrome P450 genes, *CYP4DE1* and *CYP6CW3v2* have demonstrated increased expression when exposed to the insecticide. Targeting the two genes led to decreased expression of mRNA in the strain and increased susceptibility to the insecticide (Elzaki et al., 2015). Targeting of developmental genes and resistant genes such as *Sad* and *CYP4DE1* and *CYP6CW3v2* genes can help in developing a dsRNA-based pest management in the planthopper.

The Asian citrus psyllid (*Diaphorina citri* Kuwayama) is an economically important pest of citrus and is known to damage the plant by feeding on the sap of citrus plants. It also is a carrier for the bacteria causing citrus greening disease, *Candidatus liberibacter asiaticus*. Due to this dual threat, insecticides are used profusely to curb this menace. However, this has led to the pest developing resistance to the commonly used organophosphates and carbamate-based insecticides. A study by Kishk et al. (2017) explored the role of acetylcholinesterase (*AChE*) on the mortality of *D. citri* and its susceptibility to the insecticides. Introduction of dsRNA-*AChE* into the insect system led to increase in mortality rate of both nymph and adult. However, the silencing led to only selective susceptibility of the insect species while it remained tolerant to a few insecticides. Among the many detoxification enzymes found in hemipterans, UDP-glycosyltransferases (UGTs) play an essential role in coping with insecticides in *D. citri*. A few UGT genes are identified and their downregulation was studied in correspondence to insecticide susceptibility. RNAi knockdown of the four such identified genes led to increase in susceptibility to the insecticide, imidacloprid (Tian et al., 2019). Therefore, in *D. citri*, similar genes may be identified to understand their relation with insecticide resistance and their management strategies.

## Rnai in Lepidopterans

Unlike its counterparts, this class of insects has seen major variation in efficiency of dsRNA delivery and consequential RNAi. In insects, small RNAs play essential roles in several cellular pathways, transcriptional and translational mechanisms and gene expression. In lepidopterans, apart from the generic microRNAs (miRNAs), small-interfering RNAs (siRNAs) and PIWI-interacting RNAs (piRNAs), small non-coding RNA (ncRNA) have also been identified. In a study carried out in *Bombyx mori*, genes from the three major RNAi silencing pathways, namely, miRNAs, siRNAs, and piRNAs were observed to be involved in silencing processes (Nie et al., 2013). Lepidopterans in general have reduced susceptibility for gene silencing due to the alkaline gut environment, which does not allow the dsRNA to reach the epithelium. In addition, there is an array of nucleases which degrades the dsRNA when it reaches the gut. The first successful attempt of using RNAi against a lepidopteran was in 2007 against *Helicoverpa armigera*, targeting a monooxygenase gene necessary for detoxifying gossypol from cotton. Additionally, dsRNA delivered through a transgenic plant-mediated method has led to increased mortality and thus, an optimized protocol is being explored to develop a successful pest control mechanism (Kolliopoulou and Swevers, 2014). Limited success has been obtained in *in vivo* RNAi in lepidopterans, especially in the larval stages, which mostly feed on plants (Li et al., 2015c).

The initial studies carried out in lepidopterans were in 2002 in three separate species. Quan et al., 2002 observed knockdown of a pigmented gene in *B. mori* embryos. A pattern recognition protein, hemolin, was targeted in *Hyalophora cecropia* embryos by Bettencourt et al. (2002) and in *Spodoptera litura* larva a *Bacillus thuringiensis* toxin receptor was successfully targeted (Rajagopal et al., 2002). After this, advancements were made in RNAi experiments in other species of lepidopterans (Table 1). As has been mentioned earlier, significant variation has been observed.

It has been determined that insects lack the RNA-dependent RNA polymerase (RdRP) enzyme, further indicating that lepidopterans lack silencing amplification circuits centred around siRNA production. Systemic RNAi, which is the silencing of gene after introduction of dsRNA into the insect system, would not be possible in lepidopterans. Keeping this in context, researchers aimed to study the effect of spraying dsRNA on insect body surface to determine whether it is able to exhibit an RNAi effect. In a study pertaining to this concept, a hexameric storage protein gene (*OfSP*) was identified in *Ostrinia furnacalis* and the dsRNA for major domains was synthesized and sprayed on lepidopteran species *Ostrinia furnacalis* and *H. armigera*. The *OfSP* gene protein is methionine-rich and has high expression at the larval stage. It consists of three functional domains N, M, and C and the dsRNAs for the domains were sprayed on the lepidopteran species. In order to analyse the effect of spraying on the species, a 21 nt siRNA library was constructed. As a result of this, systemic RNAi was observed leading to ecdysis of larval and pupal stages, and sterility of eggs produced. The discovery provided concrete leads for broad-spectrum bio-insecticides. Also, it was seen that a combination of domains exhibited varied results in both the species implying a possibility of species-specificity (Zhang et al., 2015b).

It has been well established that the uptake of dsRNA in lepidopterans is limited as compared to the other insect species; however, it has also been observed that lepidopterans are comparatively more susceptible to viral infections. These infections hinder the RNAi machinery by producing viral suppressors of RNAi (VSR) which inhibit uptake (Swevers et al., 2013). In order to verify this theory, several lepidopteran cell lines have been studied to understand the physiological processes underlying infection of viral infection and their impact on RNAi machinery. In one such study, the RNA viruses Flock house virus (FHV) and Macula-like virus (MLV) were used for this purpose and latent persistent viral infections were observed in certain cell lines. In certain cell lines of *B. mori*, *Trichoplusia ni* and *Spodoptera littoralis*, the cell lines were found to be uninfected with the virus and RNAi reporter assay demonstrated that a RNA hairpin structure formation protected the RNAi machinery from the VSR genes activity. Additionally, persistent viral infections in the cell lines showed that the VSR gene from both the viral species remained intact in spite of uninhibited RNAi machinery. This implied that protection against RNAi is vital for the survival of the virus (Swevers et al., 2016). In another related study conducted by Hodgson et al. (2019), the B2 RNAi suppressor gene from FHV was expressed in baculovirus and was combined with dipterans and coleopterans. This study aimed at studying the effect of B2 in dicer-2-mediated RNAi activity. The B2 gene encodes a VSR in FHV and inhibits the initial steps of siRNA pathway by binding to the dsRNA molecule. Baculovirus expressed B2 was able to inhibit the endogenous dicer-2 activity, but only at higher expression levels. It was concluded in this study that introduction of a stronger promoter and studying the production of other proteins co-expressed with B2 would help further examine the effect on dicer-2 activity.

In order to explore the other factors which impact the RNAi efficiency, (Shukla et al., 2016) studied the dsRNA domain components and dsRNA degradation. Interestingly, upon injection of dsRNA into coleopteran (*L. decemlineata*) and lepidopteran (*Heliothis virescens*) cell lines, a siRNA band was observed in the former whereas degraded dsRNA was obtained from the haemolymph of the latter. This indicated an inability of the dsRNA to be processed onto siRNAs in lepidopterans, which was a significant discovery taking into perspective the poor RNAi response. A study on the lepidopteran species, *Spodoptera litura*, showed the role of multiples dsRNAses (nucleases) present in the gut of larva. Four such nucleases were identified which were able to collectively degrade dsRNA and was upregulated especially during the feeding. Multiple dsRNAses coded by the *S. litura* genome were found to be responsible for the refractory nature of lepidopterans towards RNAi (Peng et al., 2020). In addition to this, the movement of dsRNA within the cells was found to play a major role in varied efficiency. dsRNA fed to both the species was detected in the peripheral and fat tissues of *H. virescens*. On the other hand, siRNA was detected in the gut, fat tissues and epidermis of *L. decemlineata* and not *H. virescens*. Targeted knockdown using specific dsRNA showed that *L. decemlineata* was able to completely reduce the gene expression while *H. virescens* was unable to do so for the same. Apart from transportation being a factor for inefficient RNAi machinery, reduced accessibility of the dsRNA within the RNAi machinery was also deduced to play a significant role in the reduced activity in lepidopterans (Shukla et al., 2016). Similar studies with different lepidopteran species have been carried out in comparison to coleopterans, providing robust evidence of the instability of dsRNA (Guan et al., 2018).

The inaccessibility of dsRNA and its associated instability have been related to specific nucleases present in different tissues of the species which do not allow persistence in the insect system. RNAi efficiency-related nuclease (REase) studied in *Ostrinia furnacalis* is encoded by the gene *up56*. This gene had been found to be responsible for the refractory nature of lepidopterans to RNAi. Expression profile studies demonstrated that in the presence of dsRNA, *up56* is upregulated. Decreasing the expression of REase was also seen to enhance the RNAi machinery which directly implicated towards its role. Interestingly, REase is also known to be a competitive inhibitor of dicer protein and hence the inability to process the dsRNA into siRNA. Homologs of this protein have been determined in other lepidopteran species such as *H. armigera* and *T. castaneum* and it was found that REase is found exclusively in lepidopterans (Guan et al., 2018). Characterization of this protein showed that it belonged to the PIN family of proteins which are predominantly nucleases; other PIN domain proteins have been known to play a regulatory role in yeasts and *C. elegans* (Domeier et al., 2000).

Taking these factors into account, various methods have been explored in order to understand the underlying mechanisms of efficacy of RNAi in lepidopterans as well as alternate methods to overcome identified challenges. Plant-mediated RNAi technology (PMRi) has immense potential against pest control; however, its effectiveness has yet to be explored under field conditions and in recalcitrant insect species like lepidopterans. However, certain successful trials have been obtained in coleopteran, hemipteran and lepidopteran species by engineering host plants to produce dsRNA. For instance, *Manduca sexta* and *M. quinquemaculata* are two species dominantly feeding on *Nicotiana* sp., wild and cultivated. Transgenic *Nicotiana* sp. containing dsRNA targeting mid-gut expressed genes of *M. sexta* showed strong silencing ability. The genes targeted were nicotine-ingestion induced cytochrome P450 monooxygenase and lyciumoside-IV-ingestion induced β-glucosidase1 which primarily function in the larval stages for response against ingestion of host toxins. The transgenic *Nicotiana* was also able to silence homologous genes in *M. quinquemaculata* and the expression of the genes were reduced significantly. This study also implied towards the ability of PMRi technology to be able to function against congeneric species, species belonging to the same genus (Poreddy et al., 2017).

It is noteworthy that most of pest infestations occurring in agricultural fields are due to lepidopterans species pests. However, a lack of pest control methods gives rise to the need for robust mechanisms for pest management. RNAi technology is the most widely used tool for this purpose, though variable efficacy has led scientists to come up with supporting strategies. The primary objective undertaken is to increase the stability of the dsRNA in the biological system of the insect and prolong its activity. As has been previously stated, the alkaline pH of the gut of lepidopterans leads to degradation of the dsRNA. Thus, protective compounds have been explored to form a complex with dsRNA and hence prevent degradation. Several polymers have been utilized for this purpose, such as chitosan (Zhang et al., 2010). Polymers with basic side residue are known to help in reduced decomplexation of dsRNA at alkaline pH such as guanidine residue. A study was carried out in *Spodoptera exigua*, wherein a complex of dsRNA with guanidine polymer was developed, and monitored for uptake efficiency, stability and subsequent RNAi machinery activity. Surprisingly, at pH as high 11 the guanidine residue was able to prevent degradation of dsRNA for upto 30h in *S. exigua*. In addition, an enhanced cellular uptake mechanism was seen using microscopy techniques. Finally, guanidine containing polymer-nanoparticles saw a more improved RNAi effect wherein compared to the naked dsRNA activity of 16%, polymer protected dsRNA was 53% (Christiaens et al., 2018). This study formed robust conclusions on the application of nanotechnology integrated with RNAi strategies to obtain a superior pest management program. In a study on similar lines, Parsons et al., 2018 formulated a cationic polymer of guanidine, Polymer-dsRNA interpolyelectrolyte complexes (IPECs) which was found to be able to effectively take up dsRNA and also protect it from the nucleolytic activities of the *Spodoptera frugiperda*. Upon feeding to larvae, increased larval mortality and gene-specific knockdown were observed.

Improvement in RNAi efficiency has also been attempted by enhancing dsRNA abundance. This has been achieved by developing concatemerizing dsRNAs (C-dsRNA) targeting Acetylcholinesterase of *Plutella xylostella*. In comparison to non-concatemerized long dsRNAs, the former showed high target gene silencing, lowered insect weight and high mortality. These results of concatemerizing was associated with enhanced production of *Dicer* and *Ago* proteins which govern gene silencing machinery. The short repeats of C-dsRNA probably also reduced the possibility of off-targets which are prominently found in long dsRNAs (Sharath Chandra et al., 2019). The results demonstrated the potential of this technology in reinforcing RNAi silencing activity and further studies would unquestionably deliver powerful pest management tools.

Another aspect which has been taken into consideration is the regulation of genes involved in the RNAi pathway. Wang et al. (2018a), knocked out four genes responsible for functioning of the nervous system in *Helicoverpa zea*. The genes identified are orthologous to genes in *D. melanogaster* and encode neuronal channels which are insecticide target sites. dsRNA corresponding to these genes was synthesized and delivered to *H. zea via* various methods, *viz.*, microinjection into eggs, soaking of eggs and feeding the larva. Discernible results for induced RNAi were obtained only in the dsRNA microinjected eggs which provided conclusive evidence for the importance of the genes identified in the RNAi machinery. The researchers however, noted the failure of the latter two modes of delivery and provided explanation saying that it could be attributed to the nucleases present in the mid-gut of the species. Soaking of eggs to induce dsRNA uptake has been performed in several insect species and has been successfully carried out specifically in the lepidopteran species *Ostrinia furnacalis* (Wang et al., 2011). The difference in uptake efficiency has been suggested to be due to variation in structure and permeability of egg chorion. Nevertheless, the study did portray certain promising avenues in gene knockout studies for induced RNAi machinery and to overcome target site resistance (Wang et al., 2018a).

Lepidopterans have demonstrated recalcitrance to RNAi and have baffled researchers due to their wide variation across species. However, in spite of this, the core RNAi components remain conserved such as the Dicer protein. Li et al. (2015b) had demonstrated that over expression of *Ago2* gene enhanced the dsRNA mediated RNAi in *B. mori*. Over expression of *Dicer2* has been further studied. The Dicer protein of *B. mori*, *BmDicer2*, was taken for this purpose and over expressed. It was co-expressed with *Ago2* to see if this would have a synergistic effect on RNAi efficiency. *BmDicer2* over expression showed longer lasting silencing effects even in comparison to over expressed *Ago2* which implied that RNAi silencing was dependent more on the amount of siRNA as compared to the siRNA loaded in the RISC complex. The study also provided conclusive evidence that combining multiple strategies could be a promising approach as co-expression of the two proteins increased the susceptibility of *B. mori* to dsRNA uptake. Expression of *Dicer2* facilitates the synthesis of siRNA while *Ago2* facilitates its loading onto the RISC complex (You et al., 2020). The complementarity of the functionality of the proteins is the key cause of the above results. Evidently, multiple strategies have shown to be able to have a greater impact on RNAi competency. Prentice et al., 2019 adopted a similar mechanism in which *Dicer2* was over expressed and gut nucleases were downregulated in a coleopteran species, *Cylas punctiollis*. This study also shed light on the use of loss-of-function for effective RNAi in pest management which can be extrapolated to lepidopteran species as well.

## Strategies For Efficient Rnai Application

The feasibility of RNAi technology has been widely accepted, however, there are a few challenges often encountered during the usage of the technology. Primarily, the issues lie during dsRNA uptake and stability and recalcitrance of certain insect species (Zhang et al., 2013). Systemic RNAi refers to the suppression of mRNA from target gene as a result of dsRNA *via* various delivery techniques such as microinjection, cell soaking or feeding. The absence of a functional systemic RNAi arrangement would lead to inefficiency in silencing mechanism. However, there may be some differences in the conserved nature of these components which leads to variation in dsRNA uptake across insect species (Joga et al., 2016). This is evident from the differences across various insect orders as mentioned above. Systemic RNAi technology is of two kinds: cell-autonomous RNAi and non-cell autonomous RNAi (Huvenne and Smagghe, 2010). Cell autonomous RNAi refers to RNAi which occurs inside the cell while non-cell autonomous RNAi occurs after silencing signals are triggered from one cell/tissue to another. Non-cell autonomous RNAi is triggered by environmental exposure such as soaking and injection. The non-cell autonomous systemic nature of insect species also contributes to the difference in dsRNA uptake.

**Figure 2:**
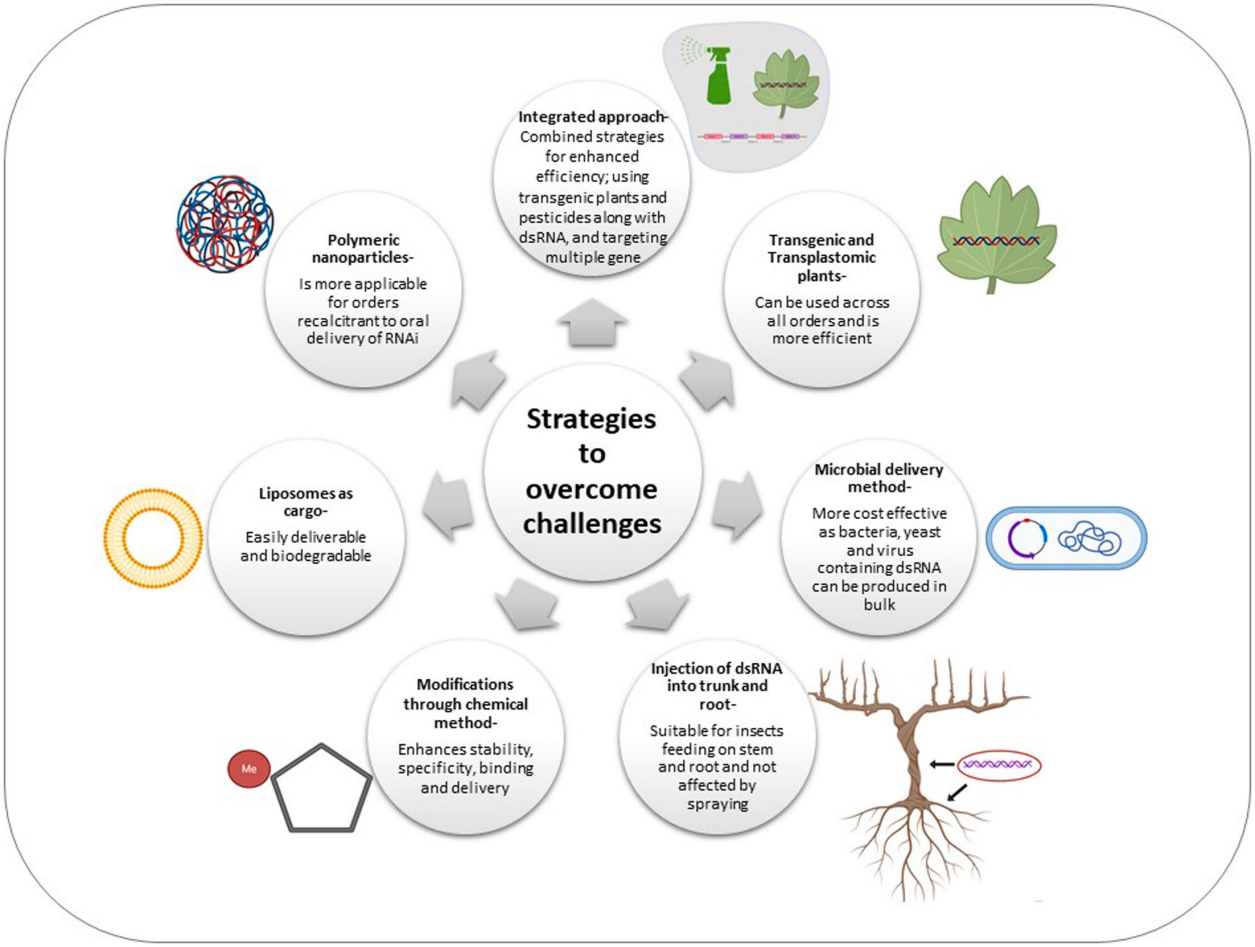
Schematic representation of various strategies adopted to overcome the challenges of RNAi across insect orders.

Taking the above in perspective, novel technologies have been developed which can be used in an integrative manner in order to improve the efficacy of RNAi (Figure 2).

## Nanoparticles

Polymeric nanoparticles have been used extensively owing to their stability and ease for modification along with environmental safety. These particles facilitate the transport of dsRNA across the gut epithelial tissues and also protect the dsRNA from nuclease degradation (Kunte et al., 2020). Chitosan polymers, for example, have been used in different insects and the target genes were found to be effectively knocked down in all the species. Target genes *AgCHS1* and *AgCHS2* and *semala* were knocked down in *A. gambiae* and *A. aegypti* successfully (Zhang et al., 2015c), while fluorescent nanoparticle and dsRNA was fed to *Ostrinia furnacalis*. In the latter, clear RNAi silencing was observed with reduced larval size and eventual death (He et al., 2013). The effects of nanoparticle mediated dsRNA delivery are evident in insect species which are recalcitrant to oral RNAi delivery, such as lepidopterans. Guanylated nanoparticle coated dsRNA targeting chitin synthase was used in lepidopteran species *Spodoptera exigua* and 53% increase in mortality was observed as compared to naked dsRNA delivery in the species (Christiaens et al., 2018). The feeding dsRNA tethered CQDs (Carbon Quantum Dots) increased RNAi efficiency in *B. tabaci* compared to naked fed dsRNA (Kaur et al., 2020). Further, these studies suggested that targeting genes like *HSPs (Heat shock proteins)*, *Knottin* and *Cyclophin* involved in virus transmission by *B. tacaci* through tethering their respective dsRNA with CODs hampered the virus titre in the vector. Another refractory species *Acyrthosiphon pisum*, belonging to Homoptera was fed dsRNA with BAPC targeting an important transcript required for survival. Premature death of the aphid was observed (Avila et al., 2018). The nanoparticles used to date, as mentioned above, contain amino acid with pKa values ranging from 9 to 13. Such nanoparticles are more stable in neutral to alkaline gut environment and thus, also impart stability to the dsRNA once ingested by the insect (Kunte et al., 2020).

The dsRNA is then most likely internalised by the cell through endocytosis after binding to the cell membrane (Li et al., 2013b). However, the exact mechanism pertaining to the movement of nanoparticle coated dsRNA is not known clearly. To understand the transport of nanoparticles, fluorescent labelled dsRNA was complexed with BAPC in *T. castaneum* and the midgut, epithelial tissues and malphigian tubules were studied for traces of fluorescence. It was deduced that dsRNA associated with BAPC was taken up by mid gut as compared to naked dsRNA where no such fluorescence was seen (Avila et al., 2018).

Considering the issue with stability and short application window of topically applied dsRNA, clay nanosheets as a carrier for dsRNA have been explored. In this, dsRNA is loaded onto non-toxic, degradable and layered double hydroxide (LDH) nanosheets. These LDH nanosheets carrying the dsRNA were found to sustain for longer durations (30days) on the plant surface without washing off and demonstrate excellent efficiency against plant virus. LDH nanosheets or BioClay have been tested effectively in *N. tabacum* plants against CMV and the uptake of dsRNA through leaf surfaces has been assessed through GUS reporter assay. The study provided insight into the topical application of dsRNA using clay nanosheets for systemic uptake of dsRNA and targeting of potential insect pests which behave as carriers of various plant viruses (Mitter et al., 2017).

## Liposomes

Liposomes are formed from non-toxic lipid molecules and are used for delivery of cargo molecules. They are easily deliverable, have minimum side-effects and are biodegradable. Certain dipterans like *D. melanogaster* lack the homologous gene used for Sid-2 and depend on endocytosis-mediated dsRNA uptake which is a time consuming process (Saleh et al., 2006). An experiment was conducted using four different species where cationic liposomes encapsulating dsRNA targeting tubulin exhibited RNAi silencing as compared to naked dsRNA which did not affect the insect. Also, lipofectamine was able to produce similar results in *Drosophila suzukii* and 40–50% of mRNA silencing was observed. Thus delivery using liposomes has proven to be a useful method in achieving RNAi (Taning et al., 2016).

## Modifications Through Chemical Methods

Chemical modifications can be done to improve the stability, specificity, binding affinity and delivery of dsRNAs and siRNAs. One such method is addition of a methyl group to the 2' carbon of ribosyl ring of the siRNA, which has shown a significant decrease in off-target effect. This method is also known to increase the specificity of dsRNA due to the unique functional group added (Jackson et al., 2003). Additionally, 3' overhangs, such as 3'TT, are known to show more efficiency in gene silencing as compared to blunt end siRNA duplexes (Czauderna et al., 2003). Nonetheless, the safety ethics for chemical modifications need to be considered as well as the cost-effectiveness.

## Injection Into Trunk and Root Systems

Phytophagous insects, which generally attack the root and stem of plants, are not usually affected by RNAi silencing methods through spraying. A more specific method which has been developed is treating the individual root and stem system by foliar spraying, root soaking and stem injection of dsRNA. It was observed that the plant was able to take in the dsRNA effectively and that it is taken up by the vascular system. Hemiptera *viz*, leaf hopper and psyllids, which feed on the xylem were also seen to contain the dsRNA after feeding on citrus tree and grapevine. The dsRNA which was specific for arginine kinase was able to target it efficiently and an increased mortality was observed in the hemipterans (Hunter et al., 2012).

Certain coleopterans like *Diabrotica virgifera* and *Diaprepes abbreviates* infect the plant by attacking the root system. They then feed on the leaves of the plant in addition to the female laying eggs on the plant surface post mating on the field itself. A novel way of tackling this solution was by hypothesizing that delivery of dsRNA could be done through irrigation, thereby, preventing the larva and insects at the root level itself (Andrade and Hunter, 2016). Another species which was studied in context with root adsorption as a means of dsRNA uptake and transport was in a lepidopteran species, *Tuta absoluta*. These insects feed on plants leaves and an astounding 80% of mortality was observed implying that plant phloem is capable of transporting dsRNA to all the tissues of the plant and can target insects which feed on plant tissues as well as sap fluid (Majidiani et al., 2019). The single challenge faced during such studies was the stability of the dsRNA in the soil (Joga et al., 2016).

Root soaking may not be a viable option for perennial plants and thus, trunk injection was considered as an alternative option. The phloem of a plant is primarily responsible for transport of molecules and signals, and is an RNase free environment which will help in longevity in stability of the dsRNA. Arborjet® is one such trunk injection system which has been developed for several plant species (Botton et al., 2004). Root soaking requires the uprooting of the plant and consequently soaking in dsRNA solutions and then replanting. This puts the plant system under immense stress which may also lead to plant death. Trunk injection on the other hand is a hassle-free process and can be applicable to old established orchards of crops like citrus fruits, apples, pear, and peach. Moreover, targeting the phloem and xylem systems will also target sap-sucking insects belonging to Hemiptera and Homoptera. Trunk injection methods can prove to be environment friendly as compared to insecticide spraying as well as specific to the pest rather than other natural enemies and pollinators (Joga et al., 2016). While trunk injection has been an option for feeding dsRNA to hemipterans and homopterans, which are mostly sap-sucking in nature, another method which has been suggested is root uptake through hydroponics. This technique would ensure that uprooting, soaking and replanting of plants is avoided (Ghosh et al., 2018).

These methods however will have only a transient expression of dsRNA and therefore would require continuous exposure to dsRNA which may not be an entirely feasible option. Additionally, this would require mass production of dsRNA which will be an extremely expensive process.

## Bacterial, Yeast and Viral-Mediated Delivery

Use of recombinant microbes engineered to possess dsRNA targeting specific mRNA is one of the latest techniques used. Bacterial species-expressing dsRNA can be mass-produced and sprayed on the infected plant which has proven to be an easier process as compared to mass-production of dsRNA. The Colorado potato beetle (CPB) is a major pest of potato plants. In a study conducted by Zhu et al. (2011), the coleopteran was fed with engineered *E. coli* targeting five main mRNAs of the beetle. Significant loss of weight and mortality was observed in the beetle species. Symbiont bacteria were also used in a similar approach. It was observed that exogenously applied bacterial species competed with the existing microflora of the insect gut. To ensure that there is no treatment rejection, dsRNA cassettes were engineered for the actinobacterium and proteobacterium, *Rhodnius prolixus* and *Frankliniella occidentalis*. The RNaseIII-deficient bacteria allowed the stable production of dsRNA and efficient uptake in the insect gut thus initiating RNAi machinery (Whitten et al., 2016).

The activity of live and heat-inactivated bacteria was also studied in a variety of insects. A study on *Mythimna separate* indicated that live bacteria are more potent in dsRNA activity as compared to the heat-killed, with a difference of 48 and 16% mortality in larval stages, respectively. Studies performed in *P. xylostella* and *H. armigera* exhibited 50 and 68% mortality in larval stage and adult stage, respectively, when fed with live bacteria. However, no impact was observed in neonates hatched from eggs. This implied that live bacteria have a probable effect on the metamorphosis stages of the insects. In *S. exigua* both live and heat-killed bacteria had similar effects (Israni and Rajam, 2017, Vatanparast and Kim, 2017; Wang et al., 2018b). The above studies concluded variation across orders with reference to RNAi machinery, irrespective of the state of the microbe (Kunte et al., 2020).

Such studies have been carried out also in engineered yeast systems. *Saccharomyces cerevisiae* expressing dsRNA targeting the tubulin gene was also found to successfully lead to significant death of *D. suzukii* upon ingestion (Murphy et al., 2016).

Viral dsRNA delivery methods have also been investigated in numerous insect orders. For evaluating effective interfering and target sequences in plants, recombinant plant viruses can prove to be a more efficient alternative. RNA plant virus are powerful targets of RNAi response in plants. Their replication *via* dsRNA intermediates, induces a response of RNAi by the plant, thereby, generating virus-specific siRNAs. Thus, a plant RNA virus can produce dsRNAs and siRNAs and introduction of a foreign sequence into the virus genome would also lead to generation of dsRNA and siRNA specific to that sequence. Recombinant plant viruses have therefore gained pace to achieve trans-specific RNAi during interaction of host plant and potential pathogen. Plant-infecting viruses usually move around the plant through the phloem system. Thus, virus-mediated technology can be used for insects which feed on stems. Recombinant *Tobacco mosaic virus* (TMV) has been used extensively on coleopteran and hemipteran insect species such as *Bactericera cockerelli* and *Planococcus citri*. mRNA production, progeny production and fecundity were found to be decreased in the insect species when fed with recombinant viral infected plants (Khan et al., 2013, Wuriyanghan and Falk, 2013). In another study, FHV was used containing dsRNA specific for three vital genes in *D. melanogaster* and microinjected into the insect species. A 70% reduction in gene expression was observed for all the genes in comparison to the untreated insects. Furthermore, insect species also harbour a variety of other viruses, which could be engineered to express specific dsRNA and directly introduced into insect populations. Viral modes of dsRNA delivery are a promising aspect of RNAi silencing technology; however, they are also known to encode certain RNAi suppressors. There has yet to be an appropriate optimisation of this technique in terms of safety (Sliva and Schnierle, 2010).

## Transgenic/Transplastomic Plants

Transgenic plants containing dsRNA have been an incredible discovery as they are environment friendly and also highly effective. Usually, a bacterium such as *Agrobacterium tumefacians* is cloned containing the desired dsRNA and then the plant is infected with this bacterium. The bacterial genome gets integrated with the plant genome transferring the dsRNA to the plant. This has been effectively used for many plant species and used against a wide range of insect orders including Hemiptera, Lepidoptera, and Coleoptera. However, incorporation into the transgenic plants often leads to processing of the dsRNA into siRNA by the plants inherent mechanism thereby reducing the efficiency of the RNAi machinery (Poreddy et al., 2017). To overcome this challenge, expression of dsRNA in plant organelles lacking RNAi machinery is used for increased RNAi effect. In plants utilizing the inherent nuclear mechanism the dsRNA is often converted into siRNA before ingestion by the insect. This renders the process ineffective. In chloroplast expressing dsRNA, the processing of dsRNA into siRNA is avoided as they do not possess the mechanism. dsRNA processing may lead to a reduced effect of RNAi; however, the presence of long strands of dsRNA are known to be more effective for silencing. Potato plants engineered to produce actin-specific dsRNA in chloroplast were fed to CPB larva. It was seen that complete mortality occurred in the larva fed on chloroplast expressing dsRNA as compared to nuclear expressing dsRNA which in itself was a significant discovery in terms of plant-based dsRNA delivery (Zhang et al., 2015a). Similarly, expression of *acetylcholinesterase* dsRNA in *Nicotiana benthamiana* was used against *H. armigera* and 70% mortality was observed at the larval stage itself.

Although nuclear transformed transgenic plants may not be as effective as chloroplast transformed plants, there have still been significant results in maize, soybean, rice against pests lik*e Leguminivora glycinivorella*, *D. v. virgifera*, *Meligethes aeneus* and *Nilaparvata lugens* (Zha et al., 2011; Knorr et al., 2018; Wang et al., 2018b).

## Integrated Approach

An interesting approach which has been explored are “stacked treatments.” In this, two pest control approaches are executed simultaneously to enhance the effect. For example, treatment with pesticides and dsRNA delivery simultaneously. This was tried in *D. citri* which is an important pest of citrus fruits. dsRNA targeting glutathione transferase in the psyllid was combined with pesticides thiamethoxam and fenpropathrin and an increase in mortality rate was observed (Yu and Killiny, 2018).

Similarly, dsRNA usage along with transgenic plants has also been tried and tested. In this, transgenic plants expressing Bt-toxin was combined with dsRNA expression and checked for change in RNAi efficiency. Recently United States Environmental Protection Agency approved a transgenic plant, SmartStax which expresses Bt-toxin and dsRNA targeting vacuolar protein against the insect pest, Western corn rootworm. This effort saw an increase in mortality by 80–95% as well as durability (Moar et al., 2017). In a similar experiment Bt-toxin was combined with dsRNA and targeted against *H. armigera* interfering with the juvenile hormone (JH) in cotton. This study was able to delay the resistance response of the pest to *Bt* as compared to the plant expressing only *Bt*. This study paved way for challenges faced by researchers in terms of adaptive resistance (Ni et al., 2017).

Multiple dsRNA targeting multiple vital genes were also studied as a method for enhanced silencing efficiency. In *Agrilus planipennis*, dsRNA targeting two genes. *Hsp-70* and *shibire* exhibiting an improved mortality of up to 90% (Rodrigues et al., 2018). *Tribolium castaneum* was exposed to similar environment with dsRNA targeting *Bip* and *Armet* combined with BAPC nanoparticles (Avila et al., 2018). This demonstrated an increased mortality of 60% as compared to the 25% individual effect of only dsRNA.

On the contrary, researchers tried incorporating 11 dsRNAs and studied their synergistic effects in *T. castaneum*. In this case however, the individual dsRNA showed 80–90% mortality while the combined dsRNAs failed to show any amount of RNAi silencing (Ulrich et al., 2015).

Even though stacked methods of RNAi silencing have proven to be efficient in certain cases, it still needs to be extensively studied in order to comprehend the number of dsRNAs which can be cumulatively effective.

## Biosafety and Global Regulations

RNAi technology has been effectively carried out in a wide range of insect species and has produced some promising results. However, due to certain disadvantages in some newer techniques, it is essential to take into consideration its pros, cons and potential risks in order to make it commercial and ready for field trials (Yan et al., 2020).

An important biosafety aspect worth bearing in mind is any toxic effect in organisms. Due to the limited research on detrimental effects of dsRNA in the immune systems of crop plants and organisms utilized as vectors, it is critical to study any possible side-effects of this technology. There are speculations that plants containing dsRNA might not pose an issue; however, if the dsRNA has been modified to increase its uptake or reduce the effect of nucleases then they might prove to be toxic for consumption or lead to reduced crop yield (Liu et al., 2020). The stability and persistence of dsRNA and siRNA in different ecosystems need thorough evaluation. dsRNA and siRNA in their native form are degraded in soil and aquatic systems within 2–7days; however, as mentioned above, additional carrier molecules which are used to enhance stability, persist in the environment for much longer. For example, nanoparticles as carriers require further evaluation as study on their cytotoxic nature is limited and not much is known about their degradation in the environment (Yan et al., 2020). Yet another concern observed during RNAi is the potential of insect pests to develop resistance to plants containing dsRNA by enhancing dsRNA degradation, reducing dsRNA uptake and mutation of genes governing the RNAi machinery (Liu et al., 2020). Viral-mediated RNAi possesses a potentially greater concern as viruses produce RNAi suppressors providing them with an advantage when exposed to RNAi protected plants (Yu et al., 2016). The RNAi-based technology provides a wide scope of developing highly target specific dsRNA-based molecules for managing different classes of insects. The sequence-specific mode of action makes these molecules safe to above surface non-targets and soil fauna because of less persistence in soil compared to hazardous synthetic insecticides (Wang et al., 2016; Parker et al., 2019; Bachman et al., 2020; Taning et al., 2021a). dsRNA-based next generation insecticides have numerous advantages over chemical pesticides, such as RNAi-based active molecules can be designed to target various genes without changing the sequence dependent mode of action. The lethal or sub-lethal or any other physiological impairments can be achieved depending on the gene targeted in the insect-pest (Taning et al., 2021b).

The evolution of insect resistance studies has led to a huge amount of data illustrating the challenges faced by researchers. Such data will impart critical information to formulate a robust regulatory framework for ease of executing field trials along with ensuring least risk. RNAi-mediated gene silencing is categorised under genetically modified organism regulations and a white paper was released by the United States Environmental Protection Agency (US EPA) providing a risk assessment guidance of RNAi-based crops (EPA, 2013). In 2014, the European Food Safety Authority (EFSA) organised a scientific workshop to review the status of RNAi-based GM crops in the existing GM crops framework as well as need of refinements. The panel focussed on molecular characterization, environment risk and food/feed safety assessment, which essentially are the three major aspects of GM risk assessment (European Food Safety Authority, 2014). Environmental sustainability policies of European Union (EU) and EPA have always focused on replacement of contentious pesticides with eco-friendly, efficient, and economical alternatives to ensure sustainable food production. European Commission’s Green Deal strategy, named as Farm-to-Fork (F2F), also aims for sustainable and food secure society. F2F aims to reduce the use of synthetic agrochemicals, fertilizers and antimicrobials to decrease impact on non-targets and health through promotion of integrated pest management, precision agriculture and artificial intelligence to achieve greater sustainable productions. The Commission has also cited the F2F’s pesticide reduction strategy in its Biodiversity Strategy 2030, which aims to address the decline in farmland birds and non-target insects. It also proposes to increase organic farming to 25% of cultivated land by 2030 and establishment of forest for conserving insect-pest diversity. There is also a proposal of banning the use of pesticides in urban areas and use pesticides judiciously under Sustainable Use Directive’ (SUD). The EU members under this directive need to formulate and implement policy for reduction in use hazardous pesticides and broaden the base for alternative pest control strategies. To support this, the European Crop Protection Association (ECPA) has come forward with €14 billion investment towards precision agriculture, biopesticides and other pest control alternatives. Not only EU but United Nations Environmental Program (UNEP) as well as other national and international organizations are also emphasising through various policies on reducing the risk of hazardous chemicals from food as well environment as a whole. To achieve this, these organizations are suggesting development of eco-friendly and highly target specific and economical pest-management technologies. As mentioned earlier, dsRNA targets specific mRNA for regulation of gene expression. This allows for the development of specific RNAi-based products which have a comparatively safer profile than chemical pesticides as they are less mobile through soil and also less persistent. Pesticides are usually broad spectra and have the possibility of affecting even non-target species. On the contrary, specific RNAi targeting, which could be selective at first, confirms negligible off-targets owing to the availability of *in silico* tools and genomic database. It is also noteworthy that dsRNA can be easily degraded by environmental nucleases while chemical pesticides persist longer in the environment. The i5K initiative, which targets to sequence genome of 5,000 insect species, provides wide scope and opportunity by increasing the availability of genome databases for various insect-pest species for designing RNAi-based pesticide molecules. Various dsRNA designing tools help in selecting the insect-specific target sequence with negligible effect on non-target insect species (Taning et al., 2021b). The dsRNA-based sprays for insect management are not in infancy now as many companies are in process of development as well as applied for regulatory approvals.

The commercial applications of RNAi are majorly through two approaches; GM generation through in planta delivery and exogenous application of RNAi-based products. The biosafety perspective of transgenic organisms is still debated and they may follow the regulatory guidelines as in of cry toxin-based transgenic or some other robust regulatory framework may be devised for this category; however, dsRNA expressing crops for insect or virus resistance have been approved for cultivation outside Europe. The European Union, based on recommendations of EFSA has approved the import and processing of some RNAi-based plant products. EU authorization to such RNAi-based products requires complete information on risk assessment and environmental safety. Unlike present day transgenic plants like Bt-cotton, corn, and brinjal the safety assessment of RNAi-based products may not be that complex as no new protein is to be produced in these organisms. The COST Action iPlanta (iplanta.univpm.it) is one of the largest network group of researchers from Europe actively involved in application of RNAi. One of the goals of this network is to identify biosafety data requirements for risk management and assessment of dsRNA expressing plants and their products and provide its recommendations to EFSA. The EFSA Guidance Documents on risk assessment for GMOs is also applicable to RNAi-based plants; however, it considers their safety assessment trials case-by-case (Arpaia et al., 2020). Transgenic plants however, can be used to overcome the issues faced during development of resistance to RNAi by insects. Transgenic/transplastomic plants provide an inherent selection pressure as compared to the non-transformed RNAi products which impart them with rapid resistance. In spite of the advantages demonstrated by genetically modified plants, the biosafety research for NTO and evaluating the risk assessment of RNAi-based modified crops must be done. On the other hand, exogenous application of RNAi-based products is preferred because these do not integrate into the genome of the plant and also are not heritable like transgenes. However, these may be subjected to pesticide regulations and their risk assessment protocols have to be formulated based on experiences of GM RNAi crops. Exogenous application of dsRNA, as described previously, such as trunk and root injection and root uptake through hydroponics are popular methods for direct application of dsRNA. The risk associated with the RNAi-based products is broadly classified into two areas, inadvertent impact on humans and environment as a whole. Thus, there are many mandatory aspects to be considered for RNAi-based pesticide application such as use of carriers and formulations for dsRNA stability, uptake by plants, environmental persistence of formulations and finally the impact of dsRNA on non-targets. The bioinformatics will have a key role in development of RNAi-based pesticides through identification of unique target sequences as well as in biosafety of these products by *in silico* evaluation of dsRNA sequences against the NTO. However this will largely depend on the availability genomic sequences of different NTO in various sequence databases. The dsRNA-based sprays have to pass through stringent regulatory system keeping in view general human perception that it is of biotechnological origin and risk assessment cannot be generalized, rather has to be considered on a case-by-case basis. The applicability of RNAi-based products therefore, lies greatly in the consumer perception. Shaping societal acceptance through explicitly stating benefits and shortcomings of RNAi-based products would be the first step towards acclimatizing consumers as well as stakeholders of this technology (Roberts et al., 2015; Shew et al., 2017; Liu et al., 2020).

## Conclusion and Future Prospect

Exogenously applied dsRNAs have been extensively studied for pest management. It was later discovered that RNAi could be triggered also by endogenous application of dsRNA and small RNAs. The wide range of studies performed on the major orders of insects has helped to provide a broad insight into the effect of RNAi silencing on pest management strategies. Administration of dsRNA through various methods has been studied including both oral administration and microinjection. More recent delivery methods such as using transgenic plant-mediated dsRNA delivery, use of nanoparticles, microbe-mediated and chemical delivery have proven to be more efficient in ensuring the stability of dsRNA in the gut of the insect. In spite of the intensive studies done on RNAi in insect classes, as well as model insects, the efficiency of environmental RNAi is still a variable factor. There are several parameters which affect the efficiency of RNAi in insects. Some of them are elucidated below:

- Presence/absence of systemic RNAi and pRNAi: The systemic spread of RNAi to the insect and to the progeny is an integral part of efficient RNAi silencing. This has been observed in plants and nematodes and some insects. However, certain insects lack systemic RNAi ability. Due to this, the variation among the insect orders is seen in correspondence to RNAi efficiency. Additionally, as mentioned above, certain RNA binding proteins (RBP) are a contributing factor to this phenomenon. The *StaufenC* protein exclusive to coleopterans has been characterized to increase the efficiency of the RNAi silencing machinery (Yoon et al., 2018).
- Copy number of RNAi-associated genes and dsRNase activity: There is an evident distinction in the core RNAi responsible genes in different insects. Additionally, the abundance of gut nucleases present in the gut and abdomen of insects contributes to the stability of exogenous dsRNA and the subsequent processing into siRNA. The insect gut pH is also associated with dsRNA stability and it is seen that the alkaline pH of lepidopteran gut leads to its instability and inability of the epithelium to intake the dsRNA. High pH also makes the insect gut recalcitrant to oral dsRNA administration (Shukla et al., 2016; Christiaens et al., 2020).
- dsRNA uptake efficiency: The dsRNA transportation efficiency in insect is dependent on various genes such as SID-1 like genes, endocytosis, RBPs, and sRNA processing (Huvenne and Smagghe, 2010).
- dsRNA designing and specificity: This forms one of the most important criteria for determining the efficacy of silencing. The length of the dsRNA, ability to withstand nucleases, specificity to the target loci, ensuring no off-targets to non-target organism/genes, and suitability of the target are the key points to be noted while designing the dsRNA (Huvenne and Smagghe, 2010; Bolognesi et al., 2012).

RNAi-mediated insect pest management has many advantages in comparison to its counterparts. Firstly, it is an environmental friendly method and has not known to produce any toxic effects on the ecosystem. Also, owing to the extensive studies performed over the years, the mode of action is clearly known. It has a wide agronomic application as it is known to contribute in reducing viral mediated diseases as well as having the ability to enhance agronomic traits, such as grain size. Finally, it is a highly specific technology as compared to pesticides, as the insects are often known to develop resistance to it (Liu et al., 2020).

However, its potential is limited due to certain drawbacks which are yet to be overcome through further research. The variability in environmental RNAi plays a contributing factor in the discrepancies often seen in the efficiency of the machinery. Moreover, orally introduced dsRNA are not stable in many insects due to the hostile alkaline gut environment (Cooper et al., 2019). The dosage of dsRNA to be administered is also a highly empirical process as it varies among different life stages of the insect. Sometimes, partial or/and variable silencing has also been observed and therefore, targeting a vital gene should be done to ensure toxic phenotypic effect irrespective of the extent of silencing (Liu et al., 2020).

A few gaps which are yet to be filled regarding this technology are

- The persistence of the dsRNA in the environment and its subsequent effects. Naked dsRNA has low longevity in the environment; however, aided with actin, it has been seen to persist on plant systems. As foliar application of dsRNA has been known to cause stable silencing, its persistence in the environment must be considered.
- There are also the possibility of immune–responsive effects and development of tolerance to the dsRNA. Mutations occur naturally and genetic variation among insects can reduce the complementarity between dsRNA and the target gene. Likewise, mutation on core RNAi genes might also impact dsRNA processing and transportation (Liu et al., 2020).
- Additionally, the off-target effects are not predictable and needs to be refined further in order to procure an absolutely robust and smooth machinery (Heinemann, 2019). One way to overcome the disadvantage of off-targets is *in silico* examination of the corresponding dsRNA to identify any potential homologs in other NTO. It must be however noted that a single base pair change is highly unlikely to cause off-target effect on the target gene. Short stretches of sequences pose higher likeness of binding to non-target genes and therefore longer stretches of sRNAs are more effective.

The past decade has seen a tremendous leap in this technology for pest management and has helped researches find a possible solutions to global emerging pest situations. Usage of transgenic plants expressing dsRNA is a desirable strategy to attain the appropriate effect. However, strict regulatory frameworks have limited the application of this technology. The commercialization of SMARTSTAX PRO maize was a stepping stone for transplastomic-based dsRNA delivery and further into chloroplast-transformed crops. Non-transformative medium of dsRNA is a future of this machinery. Nanoparticles, foliar spraying, root drenching, stem injection and microbial mediated RNAi are some of the future alternatives. Some biotech companies are investing in developing affordable methods for producing large-scale dsRNA which would allow the approach to be cost-effective. A critical aspect for incorporating this technology into the regulatory framework is to pay attention to public concerns and address them accordingly. Furthermore, negating the potential risk factors associated with RNAi would bring this into mainstream agriculture science as is a conventional method.

## Author Contributions

All authors listed have made a substantial, direct and intellectual contribution to the work, and approved it for publication.

## Conflict of Interest

The authors declare that the research was conducted in the absence of any commercial or financial relationships that could be construed as a potential conflict of interest.

The reviewer JJ declared a past co-authorship with the authors NA, RN, and JB to the handling Editor.

## Publisher’s Note

All claims expressed in this article are solely those of the authors and do not necessarily represent those of their affiliated organizations, or those of the publisher, the editors and the reviewers. Any product that may be evaluated in this article, or claim that may be made by its manufacturer, is not guaranteed or endorsed by the publisher.
